# (2R*,4R*,7S*,10R*,12R*)-3,11,13,15-Tetra­oxa­penta­cyclo­[5.5.3.0^1,7^.0^2,4^.0^10,12^]penta­deca-5,8-dien-14-one

**DOI:** 10.1107/S1600536813023817

**Published:** 2013-09-04

**Authors:** Goverdhan Mehta, Saikat Sen, C. S. Ananda Kumar

**Affiliations:** a School of Chemistry, University of Hyderabad, Hyderabad 500 046, India

## Abstract

The title compound, C_11_H_8_O_5_, features a ‘skipped’ diene, an *anti*-bis­(epoxide) and a cyclic carbonate, all embedded in a densely functionalized [4.4.3]propellane scaffold. The crystal packing of this diepoxide is effected primarily by C—H⋯O hydrogen bonds, which link the mol­ecules into tapes along the *b *axis. Inter-tape connectivity is brought about by centrosymmetrically disposed pairs of C⋯O contacts [3.183 (4) Å] between the C^δ+^=O^δ-^ dipoles of neighbouring carbonate moieties.

## Related literature

For our report on the crystal structure of (1*s*,6*s*)-11,13-dioxa­tri­cyclo­[4.4.3.0^1,6^]trideca-2,4,7,9-tetraen-12-one, the synth­etic precursor of the title compound, see: Mehta & Sen (2011 ▶). For salient references related to the chemistry of mol­ecules, featuring two abutting 1,3-cyclo­hexa­diene (CHD) units embedded in a rigid 11,13-dioxa[4.4.3]propellane framework, see: Ashkenazi *et al.* (1978 ▶); Paquette *et al.* (1990 ▶). For references representing our own previous studies on the modes of self-assembly in oxygenated CHDs, see: Mehta & Sen (2010 ▶) and citations therein. For a reference related to *VEGA ZZ* 3.0.0 the program used to generate the MEP surface diagram of the title compound, see: Pedretti *et al.* (2004 ▶). For a discussion on graph-set analysis of hydrogen bonds, see: Bernstein *et al.* (1995 ▶); Etter *et al.* (1990 ▶). For a discussion on ring-puckering parameters, see: Cremer & Pople (1975 ▶). For details of the Cambridge Structural Database, see: Allen (2002 ▶); Bruno *et al.* (2002 ▶).
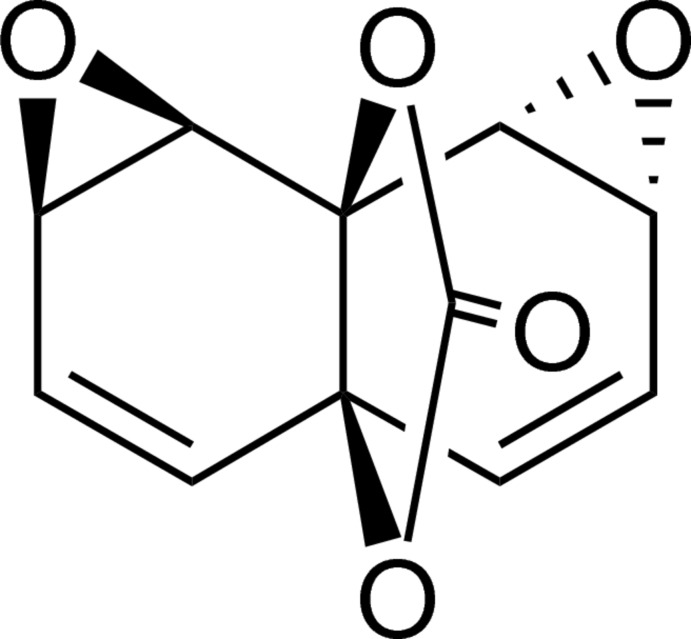


## Experimental

### 

#### Crystal data

C_11_H_8_O_5_
*M* *_r_* = 220.17
Monoclinic, 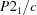

*a* = 7.170 (3) Å
*b* = 7.809 (3) Å
*c* = 16.357 (6) Å
β = 93.301 (6)°
*V* = 914.3 (6) Å^3^
*Z* = 4
Mo *K*α radiation
μ = 0.13 mm^−1^
*T* = 291 K
0.23 × 0.17 × 0.13 mm

#### Data collection

Bruker SMART APEX CCD area-detector diffractometer
Absorption correction: multi-scan (*SADABS*; Sheldrick, 2003 ▶) *T*_min_ = 0.971, *T*_max_ = 0.984
6504 measured reflections
1691 independent reflections
1319 reflections with *I* > 2σ(*I*)
*R*_int_ = 0.040

#### Refinement

*R*[*F* ^2^ > 2σ(*F* ^2^)] = 0.072
*wR*(*F* ^2^) = 0.162
*S* = 1.18
1691 reflections
145 parameters
H-atom parameters constrained
Δρ_max_ = 0.33 e Å^−3^
Δρ_min_ = −0.22 e Å^−3^

### 

Data collection: *SMART* (Bruker, 1998 ▶); cell refinement: *SAINT* (Bruker, 1998 ▶); data reduction: *SAINT*; program(s) used to solve structure: *SIR92* (Altomare *et al.*, 1994 ▶); program(s) used to refine structure: *SHELXL97* (Sheldrick, 2008 ▶); molecular graphics: *PLATON* (Spek, 2009 ▶) and *CAMERON* (Watkin *et al.*, 1993 ▶); software used to prepare material for publication: *PLATON* (Spek, 2009 ▶).

## Supplementary Material

Crystal structure: contains datablock(s) global, I. DOI: 10.1107/S1600536813023817/is5298sup1.cif

Structure factors: contains datablock(s) I. DOI: 10.1107/S1600536813023817/is5298Isup2.hkl

Additional supplementary materials:  crystallographic information; 3D view; checkCIF report

## Figures and Tables

**Table 1:** Hydrogen-bond geometry (Å, °)

*D*—H⋯*A*	*D*—H	H⋯*A*	*D*⋯*A*	*D*—H⋯*A*
C2—H2⋯O4^i^	0.98	2.53	3.509 (5)	174
C3—H3⋯O5^ii^	0.98	2.55	3.313 (4)	134
C10—H10⋯O5^i^	0.98	2.49	3.379 (4)	151

Symmetry codes: (i) 
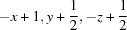
; (ii) 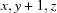.

**Fig. 1.**
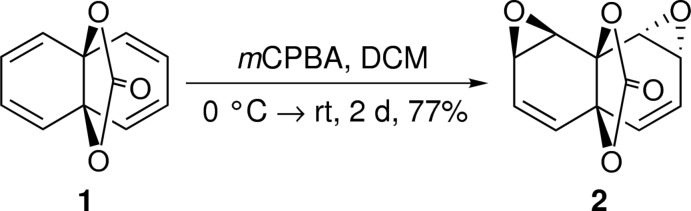
Preparation of the diepoxide 2 from the tetraene 1.

**Fig. 2.**
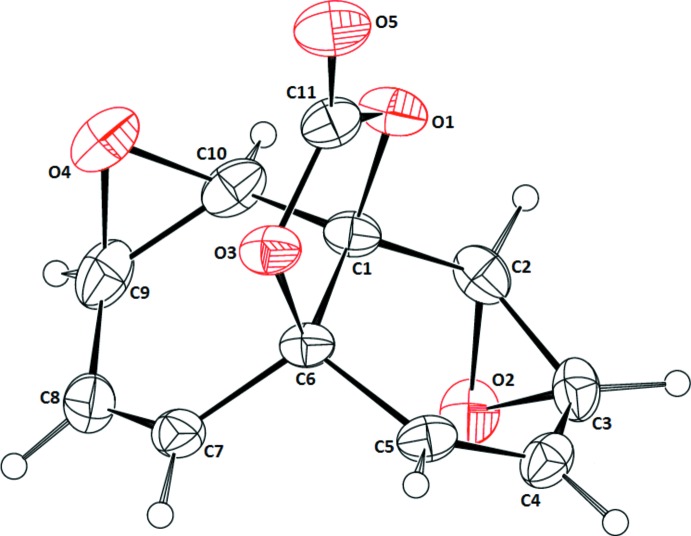
View of the diepoxide 2, with the atom numbering scheme. Displacement ellipsoids for non-H atoms are drawn at the 30% probability level. H atoms are shown as small spheres of arbitrary radii.

**Fig. 3.**
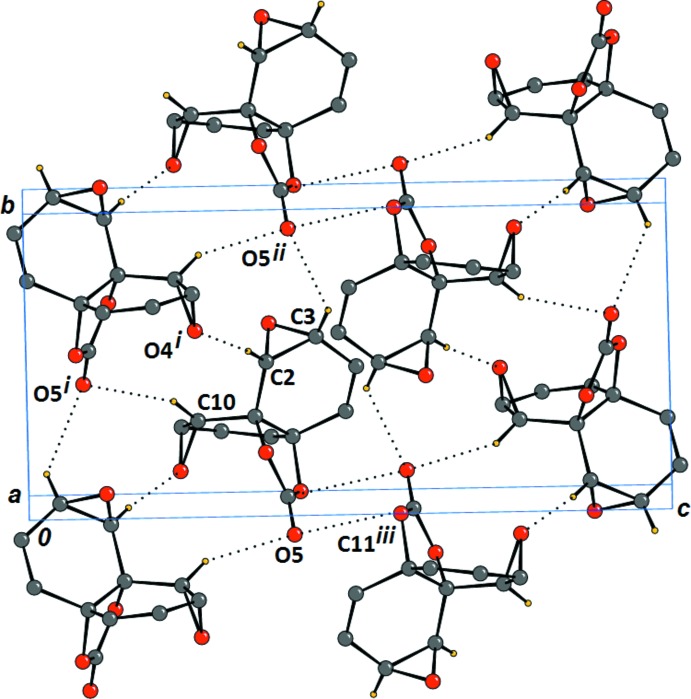
The molecular packing of the diepoxide 2. Non-interacting hydrogen atoms have been omitted for the sake of clarity. Dotted lines indicate the C—H···O hydrogen bonds and C···O short contacts. Symmetry codes: (i) –x + 1, y + 1/2, –z + 1/2; (ii) x, y + 1, z; (iii) –x + 1, –y, –z + 1.

**Fig. 4.**
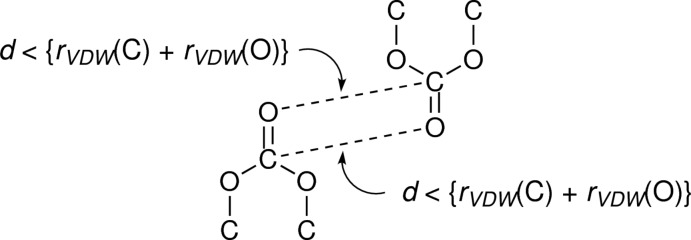
The centrosymmetric C···O interaction quadrilateral, employed in the CSD search.

**Fig. 5.**
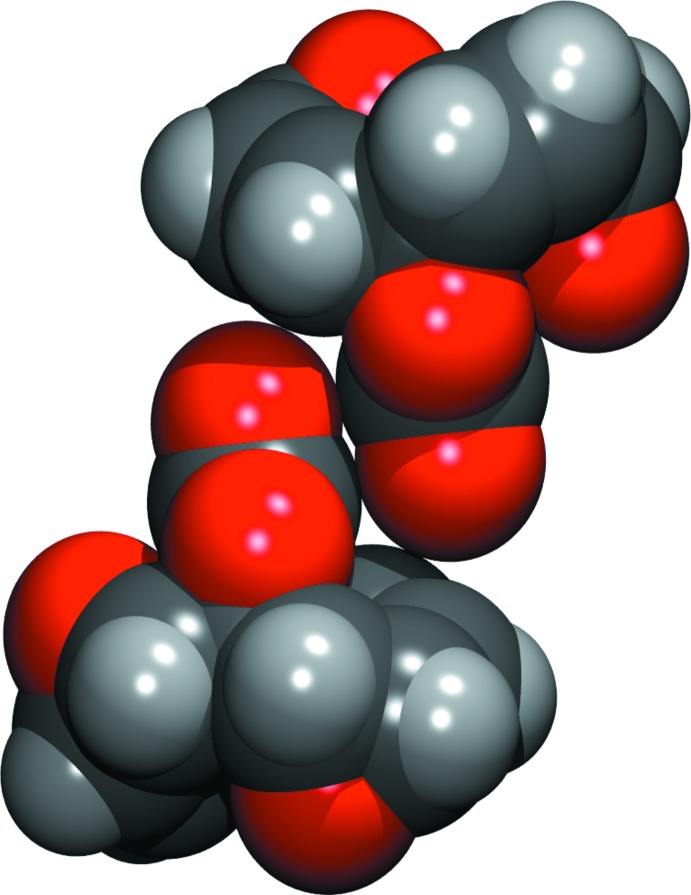
Space filling representation of two molecules of the diepoxide 2, involved in the cyclic centrosymmetric C···O interaction motif.

**Fig. 6.**
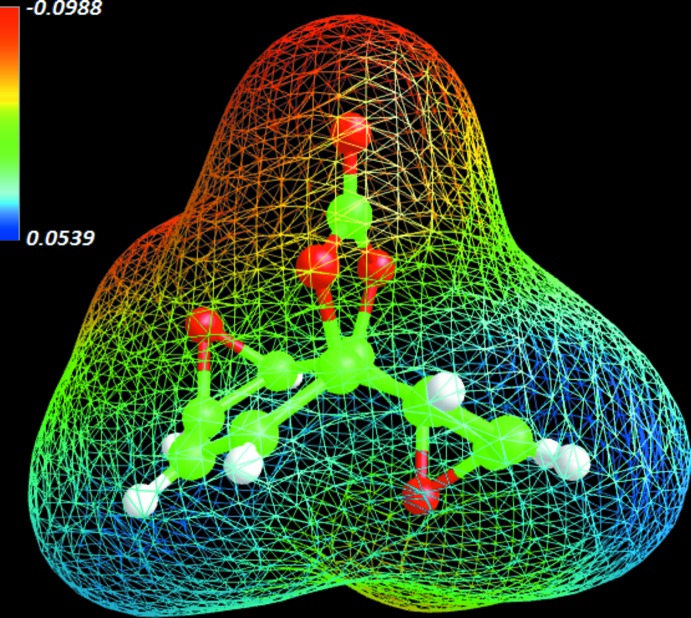
Molecular electrostatic potential (MEP) surface diagram of the diepoxide 2, as generated by VEGA ZZ 3.0.0 (Pedretti et al., 2004). The MEP calculation was performed after the semi-empirical charges were assigned by a single point MOPAC calculation, employing the MNDO method.

## supplementary crystallographic information

### 1. Comment

In a recent communication, we had introduced the C—H···O hydrogen bonded self-assembly of the crystalline tetraene 1 – the synthetic precursor of the title compound 2 (Fig. 1) – as a foil to highlight the rather singular crystal packing (lacking in any obvious hydrogen bonds) observed in two structurally related [4.4.3]propellanes (Mehta & Sen, 2011). During the course of this study, we recognized that the tricyclic carbonate 1 features a novel structural attribute – namely, two abutting 1,3-cyclohexadiene (CHD) units embedded in a rigid 11,13-dioxa[4.4.3]propellane framework (Ashkenazi *et al.*, 1978; Paquette *et al.*, 1990). In view of our on-going activity in delineating the patterns of self-assembly in oxygenated CHDs (Mehta & Sen, 2010), it was of considerable interest to investigate the diversely oxy-functionalized CHD moieties that might be accessed from the tetraene 1.

*m*-Chloroperbenzoic acid (*m*CPBA) mediated epoxidation of 1 was carried out and though sluggish, led to the formation of the title compound 2 in presence of excess peracid (Fig. 1). Routine characterization of 2 by NMR spectroscopy revealed it to be the product of an unsymmetrical *bis*-epoxidation of the *C*_2v_-symmetric 1. This was decidedly an uncanny result that was quite unpredictable and could not be readily reconciled with stereo-electronic preferences. In order to settle its stereo-structure by single-crystal XRD analysis, the diepoxide 2 was crystallized by slow evaporation of its saturated solution in 1:2 EtOAc-hexanes.

The crystal structure of 2 was solved and refined in the centrosymmetric monoclinic space group *P*2_1_/*c* (*Z* = 4). Interestingly, the two epoxy functionalities in 2 were found to have a 1,4-*anti* relationship to one another (Fig. 2). As suggested by their conformational analysis [Ring C1–C6: *q_2_* = 0.225 (3) Å, *q_3_* = 0.086 (3) Å, *φ_2_* = 322.5 (9)°, *Q_T_* = 0.241 (3) Å, *θ_2_* = 69.2 (7)°; Ring C1/C6–C10: *q_2_* = 0.201 (4) Å, *q_3_* = -0.080 (4) Å, *φ_2_* = 200.6 (10)°, *Q_T_* = 0.215 (3) Å, *θ_2_* = 111.8 (11)°], the two cyclohexene rings in 2 adopt a puckered form, somewhat intermediate between a pure twist-boat (*TB*) and a pure half-chair (*HC*) conformation (Cremer & Pople, 1975). Puckering parameters of the central five-membered ring [*q_2_* = 0.238 (3) Å, *φ_2_* = 51.9 (7)°] are close to the values expected for an ideal *C*_2_-symmetric 'twist' (*T*) conformation (Cremer & Pople, 1975).

Molecular packing in 2 was brought about primarily *via* the agency of C—H···O hydrogen bonds which linked the molecules into zigzag tapes essentially along the *b* axis (Fig. 3). Within each of these tapes, a pair of hydrogen bonds (C2—H2···O4 and C10—H10···O5), defining an *R*^2^_2_(11) motif (Etter *et al.*, 1990; Bernstein *et al.*, 1995) connected the diepoxide molecules related by the 2_1_ symmetry, while another H-bond (C3—H3···O5) consolidated the architecture by linking the molecules, translated along the *b* axis. While no inter-tape hydrogen bonds were observed, a closer analysis of the crystal packing in 2 revealed C···O short contacts [C11···O5, *d* = 3.183 (4) Å, symmetry code: –*x* + 1, –*y*, –*z* + 1] between the molecular tapes along the longest *c* axis. These C···O contacts involved centrosymmetrically disposed pairs of neighbouring C^δ^^+^═O^δ^^-^ dipoles (Fig. 3) and described an interesting cyclic dipolar interaction motif, much akin to an *R*^2^_2_(4) O—H···O hydrogen bonding loop. In fact, a Cambridge Structural Database search [CSD version 5.33 (November 2011); Allen, 2002; Bruno *et al.*, 2002] with such a C···O interaction quadrilateral involving the carbonyl group of carbonate moieties (Fig. 4), generated only 10 hits (CSD codes: ARUJEC, CHPCBO, JOKSUX, MHIQXI, QENJUP, SECPUL, SEGCAI, VECQUP, WIZQOL and XEXVEB). Even within this coterie, the observed C···O interaction motif was, in most cases, adventitious/supportive in nature and resulted merely on account of centrosymmetrically related pairs of hydrogen bonds forcing the carbonyls to approach closer to one another. It is worth mentioning at this point that the centrosymmetric C···O interaction motif in 2, though unsupported by hydrogen bonds, might owe its existence to a congenial synergy between the shape and charge distribution in the molecule. As illustrated in Figure 5, each of the two carbonyl groups, involved in the C···O short contacts, fits in a complementary lock-and-key fashion within the single accessible 'groove' (defined by the central five-membered ring and the cyclohexene, bearing the *endo* epoxy moiety) that the diepoxide 2 presents. Coincidentally, this groove also bears that 'face' of the carbonyl functionality in 2 which features a well defined C^δ^^+^═O^δ^^-^ dipolar charge separation (Fig. 6).

To summarize, we have provided herein the first report of an attempted oxyfunctionalization of the tetraene 1 and the complete structural elucidation of the diepoxide 2, obtained in the endeavour. The supramolecular structure of 2, as obtained from the analysis of its single-crystal XRD data, was found to be quite noteworthy, particularly because the carbonyl group of the carbonate moiety in 2 not only functioned as the donor in two C—H···O hydrogen bonds, but participated in a scarcely encountered cyclic dipolar interaction motif as well.

### 2. Experimental

As delineated in Figure 1, the title compound was obtained by *m*CPBA mediated epoxidation of the tetraene 1. Thus, 1 (0.188 g, 1.00 mmol) was dissolved in dichloromethane (8 ml) and solid *m*-chloroperbenzoic acid (70% purity, 0.740 g, 3.00 mmol) was added portion wise to the stirred solution, cooled on an ice-bath. Thereafter, the reaction mixture was allowed to stir while gradually warming to room temperature on its own. The progress of the reaction was monitored by thin layer chromatography. Even after allowing the reaction to proceed for three days at room temperature, the TLC profile showed no apparent change beyond the disappearance of the starting material and the formation of 2 as the predominant product. The excess peracid was therefore decomposed with saturated Na_2_SO_3_ solution and the reaction mixture extracted thrice with dichloromethane. The combined extracts were washed with saturated NaHCO_3_ solution and then dried over anhydrous Na_2_SO_4_. Evaporation of the solvent and purification of the residue by column chromatography over silica gel with 40% EtOAc-hexanes furnished the pure diepoxide 2 (0.170 g, 77%) as a colourless solid. M.p. 158 – 159 °C.

Single crystals of the diepoxide 2, suitable for X-ray diffraction, were obtained by slow evaporation of its saturated solution in 1:2 EtOAc-hexanes.

### 3. Refinement

H atoms were placed in geometrically idealized positions with C—H distances 0.93 or 0.98 Å and allowed to ride on their parent atoms with *U*_iso_(H) = 1.2*U*_eq_(C).

### Crystal data

**Table:**

C_11_H_8_O_5_	*F*(000) = 456
*M**_r_* = 220.17	*D*_x_ = 1.599 Mg m^−^^3^
Monoclinic, *P*2_1_/*c*	Mo *K*α radiation, λ = 0.71073 Å
Hall symbol: -P 2ybc	Cell parameters from 2221 reflections
*a* = 7.170 (3) Å	θ = 2.9–25.2°
*b* = 7.809 (3) Å	µ = 0.13 mm^−^^1^
*c* = 16.357 (6) Å	*T* = 291 K
β = 93.301 (6)°	Block, colorless
*V* = 914.3 (6) Å^3^	0.23 × 0.17 × 0.13 mm
*Z* = 4	

### Data collection

**Table:**

Bruker SMART APEX CCD area-detector diffractometer	1691 independent reflections
Radiation source: fine-focus sealed tube	1319 reflections with *I* > 2σ(*I*)
Graphite monochromator	*R*_int_ = 0.040
φ and ω scans	θ_max_ = 25.4°, θ_min_ = 2.5°
Absorption correction: multi-scan (*SADABS*; Sheldrick, 2003)	*h* = −8→8
*T*_min_ = 0.971, *T*_max_ = 0.984	*k* = −9→9
6504 measured reflections	*l* = −19→19

### Refinement

**Table:**

Refinement on *F*^2^	Primary atom site location: structure-invariant direct methods
Least-squares matrix: full	Secondary atom site location: difference Fourier map
*R*[*F*^2^ > 2σ(*F*^2^)] = 0.072	Hydrogen site location: inferred from neighbouring sites
*wR*(*F*^2^) = 0.162	H-atom parameters constrained
*S* = 1.18	*w* = 1/[σ^2^(*F*_o_^2^) + (0.0604*P*)^2^ + 0.6586*P*] where *P* = (*F*_o_^2^ + 2*F*_c_^2^)/3
1691 reflections	(Δ/σ)_max_ < 0.001
145 parameters	Δρ_max_ = 0.33 e Å^−^^3^
0 restraints	Δρ_min_ = −0.22 e Å^−^^3^

### Special details

**Table:**

Experimental. IR (KBr, *ν* cm^-1^) 3057, 2203, 1805, 1524, 1204, 1263, 1032, 943, 854, 758, 725, 665, 619; ^1^H NMR (400 MHz, CDCl_3_, *δ*, p.p.m.) 6.46 (dd, *J* = 8, 3 Hz, 1H), 6.34 (dd, *J* = 8, 3 Hz, 1H), 5.91 (d, *J* = 8 Hz, 1H), 5.84 (dd, *J* = 8, 1 Hz, 1H), 4.00 (d, *J* = 3 Hz, 1H), 3.85 (d, *J* = 3 Hz, 1H), 3.73 (ddd appearing as a dt, *J* = 3, 1 Hz, 1H), 3.57 (ddd appearing as a dt, *J* = 3, 1 Hz, 1H); ^13^C NMR (100 MHz, CDCl_3_, *δ*, p.p.m.) 151.7, 130.9, 129.8, 128.1, 125.4, 78.3, 76.2, 56.8, 51.8, 49.5, 46.6.
Geometry. All s.u.'s (except the s.u. in the dihedral angle between two l.s. planes) are estimated using the full covariance matrix. The cell s.u.'s are taken into account individually in the estimation of s.u.'s in distances, angles and torsion angles; correlations between s.u.'s in cell parameters are only used when they are defined by crystal symmetry. An approximate (isotropic) treatment of cell s.u.'s is used for estimating s.u.'s involving l.s. planes.
Refinement. Refinement of *F*^2^ against ALL reflections. The weighted *R*-factor *wR* and goodness of fit *S* are based on *F*^2^, conventional *R*-factors *R* are based on *F*, with *F* set to zero for negative *F*^2^. The threshold expression of *F*^2^ > 2σ(*F*^2^) is used only for calculating *R*-factors(gt) *etc*. and is not relevant to the choice of reflections for refinement. *R*-factors based on *F*^2^ are statistically about twice as large as those based on *F*, and *R*- factors based on ALL data will be even larger.

### Fractional atomic coordinates and isotropic or equivalent isotropic displacement parameters (Å^2^)

**Table:**

	*x*	*y*	*z*	*U*_iso_*/*U*_eq_	
O1	0.5301 (3)	0.1663 (3)	0.36989 (14)	0.0500 (7)	
O2	0.7967 (3)	0.5656 (3)	0.37864 (14)	0.0514 (7)	
O3	0.7693 (3)	0.0174 (3)	0.42208 (14)	0.0432 (6)	
O4	0.7672 (4)	0.0902 (3)	0.23628 (15)	0.0688 (8)	
O5	0.4863 (4)	−0.1009 (3)	0.41171 (16)	0.0638 (8)	
C1	0.6909 (4)	0.2708 (4)	0.35416 (19)	0.0373 (8)	
C2	0.6407 (5)	0.4528 (4)	0.3733 (2)	0.0447 (8)	
C3	0.6966 (5)	0.5281 (4)	0.4515 (2)	0.0468 (9)	
C4	0.7949 (5)	0.4205 (4)	0.5129 (2)	0.0445 (8)	
C5	0.8586 (4)	0.2693 (4)	0.49495 (19)	0.0400 (8)	
C6	0.8441 (4)	0.1914 (4)	0.41165 (18)	0.0340 (7)	
C7	1.0317 (5)	0.1766 (4)	0.3782 (2)	0.0430 (8)	
C8	1.0625 (5)	0.1947 (5)	0.3007 (2)	0.0538 (10)	
C9	0.9122 (6)	0.2221 (5)	0.2393 (2)	0.0593 (11)	
C10	0.7260 (5)	0.2568 (4)	0.2652 (2)	0.0511 (9)	
C11	0.5881 (5)	0.0159 (4)	0.4026 (2)	0.0442 (8)	
H10	0.6466	0.3279	0.2281	0.061*	
H2	0.5258	0.4974	0.3458	0.054*	
H3	0.6167	0.6189	0.4716	0.056*	
H4	0.8127	0.4607	0.5663	0.053*	
H5	0.9171	0.2057	0.5371	0.048*	
H7	1.1331	0.1531	0.4145	0.052*	
H8	1.1847	0.1899	0.2846	0.065*	
H9	0.9455	0.2714	0.1870	0.071*	

### Atomic displacement parameters (Å^2^)

**Table:**

	*U*^11^	*U*^22^	*U*^33^	*U*^12^	*U*^13^	*U*^23^
O1	0.0374 (13)	0.0479 (14)	0.0635 (15)	−0.0075 (11)	−0.0084 (11)	0.0113 (12)
O2	0.0623 (17)	0.0365 (13)	0.0565 (15)	−0.0047 (11)	0.0147 (12)	0.0086 (11)
O3	0.0452 (14)	0.0288 (12)	0.0544 (14)	−0.0040 (10)	−0.0083 (10)	0.0050 (10)
O4	0.090 (2)	0.0617 (17)	0.0536 (16)	−0.0063 (16)	−0.0078 (14)	−0.0158 (13)
O5	0.0650 (18)	0.0488 (15)	0.0770 (19)	−0.0260 (14)	−0.0016 (14)	0.0047 (13)
C1	0.0344 (18)	0.0367 (18)	0.0401 (18)	−0.0036 (14)	−0.0049 (13)	0.0074 (14)
C2	0.0398 (19)	0.0388 (19)	0.056 (2)	0.0083 (15)	0.0026 (15)	0.0143 (16)
C3	0.050 (2)	0.0364 (18)	0.056 (2)	0.0000 (15)	0.0219 (17)	−0.0005 (16)
C4	0.055 (2)	0.0409 (19)	0.0387 (18)	−0.0115 (16)	0.0104 (15)	−0.0048 (15)
C5	0.0436 (19)	0.0398 (19)	0.0358 (17)	−0.0093 (15)	−0.0051 (14)	0.0055 (14)
C6	0.0356 (17)	0.0268 (15)	0.0388 (17)	−0.0038 (13)	−0.0050 (13)	0.0055 (13)
C7	0.0393 (19)	0.0357 (18)	0.053 (2)	0.0008 (15)	−0.0044 (15)	−0.0055 (15)
C8	0.051 (2)	0.050 (2)	0.061 (3)	−0.0007 (18)	0.0114 (19)	−0.0129 (18)
C9	0.083 (3)	0.056 (2)	0.039 (2)	−0.006 (2)	0.014 (2)	−0.0057 (17)
C10	0.071 (3)	0.042 (2)	0.0390 (19)	−0.0004 (18)	−0.0117 (17)	0.0021 (16)
C11	0.051 (2)	0.0364 (19)	0.045 (2)	−0.0097 (17)	−0.0016 (16)	0.0011 (15)
O1	0.0374 (13)	0.0479 (14)	0.0635 (15)	−0.0075 (11)	−0.0084 (11)	0.0113 (12)
O2	0.0623 (17)	0.0365 (13)	0.0565 (15)	−0.0047 (11)	0.0147 (12)	0.0086 (11)
O3	0.0452 (14)	0.0288 (12)	0.0544 (14)	−0.0040 (10)	−0.0083 (10)	0.0050 (10)
O4	0.090 (2)	0.0617 (17)	0.0536 (16)	−0.0063 (16)	−0.0078 (14)	−0.0158 (13)
O5	0.0650 (18)	0.0488 (15)	0.0770 (19)	−0.0260 (14)	−0.0016 (14)	0.0047 (13)

### Geometric parameters (Å, º)

**Table:**

O1—C1	1.448 (4)	C4—C3	1.460 (5)
O1—C11	1.346 (4)	C4—H4	0.9300
O2—C2	1.422 (4)	C5—C4	1.305 (5)
O2—C3	1.456 (4)	C5—C6	1.490 (4)
O3—C11	1.320 (4)	C5—H5	0.9300
O3—C6	1.474 (3)	C6—C1	1.535 (4)
O4—C10	1.421 (4)	C6—C7	1.486 (4)
O4—C9	1.462 (5)	C7—C8	1.307 (5)
O5—C11	1.183 (4)	C7—H7	0.9300
C1—C10	1.495 (5)	C8—C9	1.446 (6)
C2—C1	1.504 (4)	C8—H8	0.9300
C2—C3	1.443 (5)	C9—H9	0.9800
C2—H2	0.9800	C10—C9	1.449 (5)
C3—H3	0.9800	C10—H10	0.9800
			
O1—C1—C10	108.2 (3)	C4—C3—H3	117.0
O1—C1—C2	107.1 (3)	C4—C5—C6	124.7 (3)
O1—C1—C6	102.3 (2)	C4—C5—H5	117.7
O2—C2—C1	113.7 (3)	C5—C4—C3	121.8 (3)
O2—C2—C3	61.1 (2)	C5—C4—H4	119.1
O2—C2—H2	116.4	C5—C6—C1	113.9 (3)
O2—C3—C4	116.0 (3)	C6—C5—H5	117.7
O2—C3—H3	117.0	C6—C7—H7	118.1
O3—C11—O1	111.4 (3)	C7—C6—C1	115.9 (3)
O3—C6—C1	101.0 (2)	C7—C6—C5	110.6 (3)
O3—C6—C5	106.1 (2)	C7—C8—C9	122.0 (4)
O3—C6—C7	108.3 (2)	C7—C8—H8	119.0
O4—C10—C1	116.1 (3)	C8—C7—C6	123.8 (3)
O4—C10—C9	61.3 (2)	C8—C7—H7	118.1
O4—C10—H10	115.9	C8—C9—C10	119.1 (3)
O4—C9—H9	117.1	C8—C9—H9	117.1
O5—C11—O1	122.9 (3)	C8—C9—O4	114.8 (3)
O5—C11—O3	125.6 (3)	C9—C10—C1	120.5 (3)
C1—C10—H10	115.9	C9—C10—H10	115.9
C1—C2—H2	116.4	C9—C8—H8	119.0
C2—C1—C6	115.2 (3)	C10—C1—C2	109.1 (3)
C2—C3—C4	118.3 (3)	C10—C1—C6	114.3 (3)
C2—C3—H3	117.0	C10—C9—H9	117.1
C2—C3—O2	58.8 (2)	C10—C9—O4	58.4 (2)
C2—O2—C3	60.2 (2)	C10—O4—C9	60.3 (2)
C3—C2—C1	120.8 (3)	C11—O1—C1	109.4 (2)
C3—C2—H2	116.4	C11—O3—C6	109.9 (2)
C3—C4—H4	119.1		
			
O1—C1—C10—C9	−131.7 (3)	C4—C5—C6—O3	−130.7 (3)
O1—C1—C10—O4	−61.1 (4)	C5—C4—C3—C2	11.0 (5)
O2—C2—C1—C10	−76.4 (3)	C5—C4—C3—O2	−55.9 (4)
O2—C2—C1—C6	53.7 (4)	C5—C6—C1—C10	153.6 (3)
O2—C2—C1—O1	166.7 (2)	C5—C6—C1—C2	26.0 (4)
O2—C2—C3—C4	−104.8 (3)	C5—C6—C1—O1	−89.7 (3)
O3—C6—C1—C10	−93.2 (3)	C5—C6—C7—C8	−145.4 (3)
O3—C6—C1—C2	139.2 (3)	C6—C1—C10—C9	−18.4 (4)
O3—C6—C1—O1	23.5 (3)	C6—C1—C10—O4	52.1 (4)
O3—C6—C7—C8	98.8 (4)	C6—C5—C4—C3	1.6 (5)
O4—C10—C9—C8	−102.8 (4)	C6—C7—C8—C9	−3.4 (5)
C1—C10—C9—C8	2.3 (5)	C6—O3—C11—O1	7.5 (4)
C1—C10—C9—O4	105.0 (3)	C6—O3—C11—O5	−172.8 (3)
C1—C2—C3—C4	−3.0 (5)	C7—C6—C1—C10	23.6 (4)
C1—C2—C3—O2	101.8 (3)	C7—C6—C1—C2	−104.0 (3)
C1—C6—C7—C8	−13.8 (4)	C7—C6—C1—O1	140.3 (3)
C1—O1—C11—O3	9.5 (4)	C7—C8—C9—C10	9.7 (5)
C1—O1—C11—O5	−170.2 (3)	C7—C8—C9—O4	−56.5 (5)
C2—C1—C10—C9	112.2 (4)	C9—O4—C10—C1	−112.1 (4)
C2—C1—C10—O4	−177.3 (3)	C10—O4—C9—C8	110.2 (3)
C2—O2—C3—C4	108.7 (3)	C11—O1—C1—C10	99.9 (3)
C3—C2—C1—C10	−145.7 (3)	C11—O1—C1—C2	−142.6 (3)
C3—C2—C1—C6	−15.6 (4)	C11—O1—C1—C6	−21.1 (3)
C3—C2—C1—O1	97.4 (3)	C11—O3—C6—C1	−19.8 (3)
C3—O2—C2—C1	−113.4 (3)	C11—O3—C6—C5	99.3 (3)
C4—C5—C6—C1	−20.5 (4)	C11—O3—C6—C7	−142.0 (3)
C4—C5—C6—C7	112.0 (4)		

### Hydrogen-bond geometry (Å, º)

**Table:**

*D*—H···*A*	*D*—H	H···*A*	*D*···*A*	*D*—H···*A*
C2—H2···O4^i^	0.98	2.53	3.509 (5)	174
C3—H3···O5^ii^	0.98	2.55	3.313 (4)	134
C10—H10···O5^i^	0.98	2.49	3.379 (4)	151

Symmetry codes: (i) −*x*+1, *y*+1/2, −*z*+1/2; (ii) *x*, *y*+1, *z*.
